# Effectiveness of thiopentone, propofol and midazolam as an ideal intravenous anaesthetic agent for modified electroconvulsive therapy: A comparative study

**DOI:** 10.4103/0019-5049.68371

**Published:** 2010

**Authors:** Pratibha Jain Shah, Kamta Prasad Dubey, Chhatarapal Watti, Jaya Lalwani

**Affiliations:** Department of Anaesthesiology and Critical Care, Pt. J.N.M. Medical College and Dr. BRAM Hospital, Raipur (C.G.), India

**Keywords:** Midazolam, modified electroconvulsive therapy, propofol, thiopentone

## Abstract

Modified electroconvulsive therapy (ECT) is a safe and most effective treatment modality for major depressive disorders with suicidal tendencies. For this, one must have an ideal intravenous anaesthetic agent for induction which provides rapid onset, short duration of action, attenuates adverse physiological effect of ECT, rapid recovery without adverse shortening of seizure duration and minimum rise in serum potassium. The studies in search of an ideal intravenous anaesthetic agent are limited. Aim is to compare the effect of iv thiopentone, propofol and midazolam on induction time and quality, haemodynamics, Seizure duration, recovery time and changes in serum potassium level. 90 patients of ASA I and II of either sex having major depressive illness were randomly allocated into three groups (n = 30) based on iv induction agent used. Group I, Group II and Group III patients were induced with iv thiopentone 5 mg/kg, propofol 2 mg/kg and midazolam 0.2 mg/kg, respectively. The induction time, quality of induction, haemodynamic changes, seizure duration, recovery time and change in serum potassium level were measured and analyzed by Z test. Induction was quicker in propofol group i.e., 41.03 ± 6.11 sec than in thiopentone (50.6 ± 6.32 sec) and midazolam group (77.30 ± 6.67 sec). Seizure duration was significantly shorter in midazolam group compared to propofol and thiopentone groups. Though significant rise in HR, SBP DBP was observed in all the three groups following ECT, but rise was significantly higher in thiopentone group compared to other two groups. Significantly, faster recovery was observed with propofol. Rise in serum potassium after ECT was not significant in any of the groups. Propofol is a safe and suitable intravenous anaesthetic agent for induction of anaesthesia for modified ECT.

## INTRODUCTION

Modified electroconvulsive therapy (ECT) for major depressive disorders has established its efficacy and effectiveness as an evidence-based practice. Direct ECT was first introduced by Cerletti and Bini.[1] Since then, it has continued to occupy a central place amongst treatment modalities for a large variety of psychiatric illness like severe acute depressions with suicidal tendency, acute mania, schizophrenia, catatonic psychosis and delirium where pharmacotherapy failed.[2] The technique has proved to be simple and yet certain and replaced pharmacologically produced seizure therapy (Insulin coma therapy, camphor and pentylene tetrozol induced seizure).[3]

In earlier days, for direct ECT electric shock was given directly without anaesthesia in conscious patients. Thus, the complications like bone fracture, joint dislocation, biting of tongue and tearing off of muscle fibre were frequent.[4] Further, it was not a pleasant sight to look at convulsing and frothing patients who were being held by several persons to avoid injury.

The introduction of ultra short acting intravenous anaesthetic agents and muscle relaxant particularly succinylcholine in clinical practice gave way for modified ECT with lesser complications. Still, the duration of anaesthesia, haemodynamic changes, induction/recovery time and characteristics, change in serum potassium, interaction with antipsychotic drugs, effect on seizure duration and post ECT confusion, memory loss remains matter of concern during anaesthetic management of ECT. There is always a need of ideal anaesthetic agent which has rapid, smooth induction, short duration of action, minimal effect on seizure duration, compatible with antipsychotic drugs, minimal side effect and rapid recovery.

Thiopentone has rapid smooth induction, good anticonvulsant activity, less effect on seizure duration but associated with side effects like prolonged awakening time, arrhythmias, laryngeal spasm and post ECT nausea vomiting. Besides rapid smooth induction, good anticonvulsant activity, attenuation of haemodynamic response; propofol causes rapid recovery with slight decrease in seizure duration. Midazolam also has significant anticonvulsant activity with attenuation of haemodynamic response.[5–8]

The purpose of this study was to compare the effectiveness of thiopentone, propofol and midazolam as an intravenous agent for modified ECT in view of haemodynamic parameters, induction time and quality, seizure duration, recovery characteristic and change in serum potassium in a prospective, randomized, double-blind trial.

## METHODS

After approval from institutional ethical committee and consent from patient and relatives; 90 patients of ASA I and II of either sex, aged 18-60 years scheduled for MECT were studied prospectively over one year period. Patients with a history of full stomach, major illness like T.B., bronchial asthma, drug allergy, neuromuscular disorders, acute respiratory disorder, hypertension, epilepsy, cardiovascular disease were excluded from our study. All the patients were randomly allocated by computerized randomization table into three groups of 30 each according to intravenous anaesthetic drugs administered for induction of anaesthesia in this double-blind trial. Group I, Group II, Group III received Thiopentone (2.5%) - 5 mg/kg, Propofol (1%) - 2 mg/kg, Midazolam- 0.2 mg/kg, respectively.

All the patients were kept nil orally for six hours before procedure and allowed to continue respective antipsychotic treatment till the day of procedure. Intravenous line was secured and 1ml blood in heparinised syringe was taken for baseline serum potassium measurement. Multipara (Philips MP30) was attached for monitoring heart rate, NIBP, RR, SpO_2_ and the psychiatrist was allowed to place bitemporal ECT electrodes on forehead.

All the patients were premedicated with i.v. glycopyrrolate 0.2 mg and preoxygenated for three minutes. General anaesthesia was induced with intravenous anaesthetic agent as per the group allocatedtill loss of eyelid reflex. Then intravenous succinylcholine 0.5 mg/kg was administered to all the patients for neuromuscular relaxation. When fasciculations subsided and adequate neuromuscular relaxation obtained, adequate size Guedel’s airway was inserted to prevent tongue bite and brief pulse stimulus (90-120 volts MECT) for about 2 msec was given to produce seizure. Subsequently, all the patients were ventilated with 100% oxygen at the rate of 12 breaths per minute until spontaneous breathing returned and patients were fully recovered clinically. Second blood sample was obtained 2 minutes after ECT for serum potassium measurement. All the patients were monitored for changes in haemodynamics HR, SBP, DBP, arterial oxygen saturation, ECG changes and respiratory rate throughout the procedure. Besides induction time (i.e., from time of injecting intravenous anaesthetic agent to loss of eyelash reflex) and quality of induction; seizure duration, side effects, complications and change in serum potassium from baseline were also recorded in all the three groups. Duration of recovery (Cognitive, orientation and neuromuscular co-ordination) was recorded from injection of Intravenous anaesthetic agent to time taken to obey verbal commands opening of eye, ability to sit unaided and meet discharge criteria [Table 1].

**Table 1:** Discharge criteria post-anaesthetic discharge scoring system

Category	Description of status	PADSS score*
Vital signs	Within 20% range of pre-op value	2
	Within 20% to 40% range of pre-op value	1
	>40% range of pre-op value	0
Respiratory status	O_2_ saturation >94% on room air	2
	O_2_ saturation >94% on nasal prongs @ 4 LPM or less	1
	O_2_ saturation >94% on FM @ 10 LPM or less	0
Nausea and vomiting	Minimal, treated with oral medications	2
	Moderate, treated with parenteral medications	1
	Continues after repeated treatments	0
Pain	Acceptable to patient (with oral medications)	2
	Pain somewhat acceptable to patient	1
	Pain not acceptable to patient	0

* A minimum score of 7/8 (and/or return to same preoperative status) is achieved prior to transferring the patient to a Phase III recovery area or home (Earlier minimum score of 9/10 was there in Post-anaesthetic discharge scoring system (PADSS) but in the present study, category of surgical bleeding has been omitted as there was no need of this category).

The collected data was entered in the master chart. The results were analyzed statistically by percentage, mean, standard deviation and Z tests.

## RESULTS

The demographic characteristics as shown in Table 2 were comparable in all the three groups, Maximum number of patients were schizophrenic males in all the three groups.

**Table 2:** Demographic profile, induction time, seizure duration and rise in serum k after ECT among three groups

Parameters	Group I	Group II	Group III
Age (yrs)	30.4 ± 10.55	27.33 ± 8.06	30.23 ± 10.36
Sex (M/F)	24/6	27/3	24/6
Weight (kg)	51.8 ± 5.93	53 ± 5.92	51.4 ± 6.54
Duration of induction (sec.)	50.6 ± 6.82	41.03 ± 6.11	77.3 ± 6.67
Seizure duration (sec.)	36.26 ± 4.83	26.36 ± 2.79	19.73 ± 3.63
Rise in serum K+ after ECT mmol/L	0.3	0.2	0.2

Mean duration of induction was shortest with Group II as compared to Group III and Group I (*P*<0.001, highly significant) as shown in Table 2. Induction of anaesthesia was smooth with propofol and midazolam compared to thiopentone.

**Figure 1:**
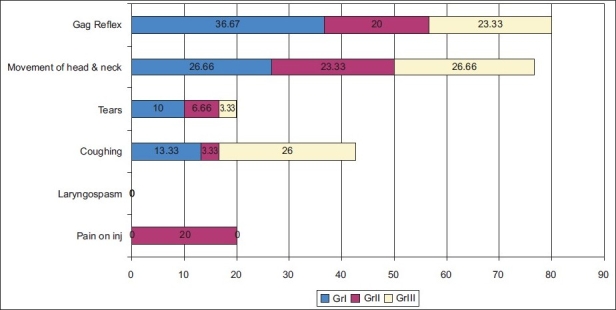
Incidence of side effects during induction (%)

Incidence of gag reflex (36.67%), coughing (13.33%) tearing (10%) during induction of anaesthesia was very high in thiopentone group as compared to propofol (20%, 3.33%, 6.66%) and midazolam group (23.33%, 0%, 3.33%) [Figure 1].

After application of ECT, significant rise in HR, SBP, DBP was observed up to 3 mins in all the three groups, but these were highly significant in thiopentone group [Tables 3 and 4].

**Table 3:** Mean heart rate at various time intervals (BPM)

Time interval (Mins)		Group I Mean ± SD	Group II Mean ± SD	Group III Mean ± SD
Basal		82.3 ± 4.25	84.53 ± 4.27	81.77 ± 4.11
After induction		85.07 ± 5.44	84.70 ± 5.59	84.30 ± 5.7
After ECT	1	120.23 ± 9.88	109.36 ± 7.83	107.66 ± 9.13
	2	124.76 ± 8.26	107.2 ± 6.99	106.3 ± 8.23
	3	119.3 ± 7.45	103.1 ± 7.1	104.2 ± 8.32
	5	99.33 ± 6.77	90.23 ± 6.69	91.33 ± 6.66
	10	88.36 ± 6.21	88.99 ± 5.43	86.2 ± 6.23
	20	87.66 ± 6.66	85.4 ± 6.69	82.44 ± 6.82
	30	87 ± 6.63	84.57 ± 6.23	81.33 ± 6.32

**Table 4:** Mean systolic and diastolic blood pressure at various time intervals (mmHg)

	Systolic BP			Diastolic BP			
	I	II	III	I	II	II
Basal	124.3 ± 6.83	123.9 ± 7.33	122.3 ± 6.78	76.7 ± 4.43	78.5 ± 4.63	81.23 ± 4.83
After induction	121.03 ± 8.42	120.9 ± 7.13	120.9 ± 7.73	79.23 ± 6.21	81.2 ± 5.53	82.23 ± 6.13
1	158.23 ± 11.81	134.22 ± 9.28	133.58 ± 10.36	99.23 ± 10.26	90.66 ± 7.53	91.36 ± 7.35
2	146.24 ± 11.61	130.66 ± 8.36	132.2 ± 9.53	93.36 ± 7.82	85.66 ± 7.76	86.3 ± 7.67
3	138.2 ± 11.66	124.33 ± 8.11	120.6 ± 8.68	87.23 ± 7.36	81.12 ± 4.95	80.66 ± 4.95
5	128.21 ± 8.30	122.67 ± 6.67	126.7 ± 6.97	83.2 ± 5.67	78.7 ± 4.62	80.7 ± 4.89
10	126.67 ± 8.28	122.36 ± 7.68	116.8 ± 7.23	79.2 ± 4.36	78.23 ± 4.12	78.32 ± 4.78
20	122.22 ± 7.88	121.22 ± 6.98	121.3 ± 7.96	77.86 ± 4.12	76.1 ± 4.26	77.22 ± 4.21
30	121.22 ± 7.82	121 ± 6.25	120.36 ± 6.96	77.36 ± 3.96	75.23 ± 3.89	76.9 ± 4.12

Though the seizure duration was shortest in Group III, the decrease in duration of seizure was highly significant in propofol and midazolam compared to thiopentone (*P* < 0.001) [Table 2].

Serum potassium was raised in all the three groups following ECT, which is not statistically significant (*P* > 0.05) [Table 2].

**Figure 2:**
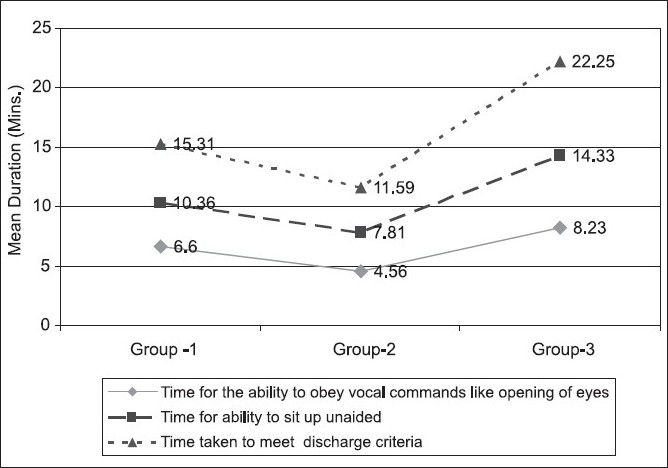
Duration of recovery indicative of cognitive, orientation and neuromuscular coordination (minutes)

The recovery of cognition, orientation and neuromuscular coordination was significantly fast in Group II (P < 0.05) followed by Group I and Group III [Figure 2].

**Figure 3:**
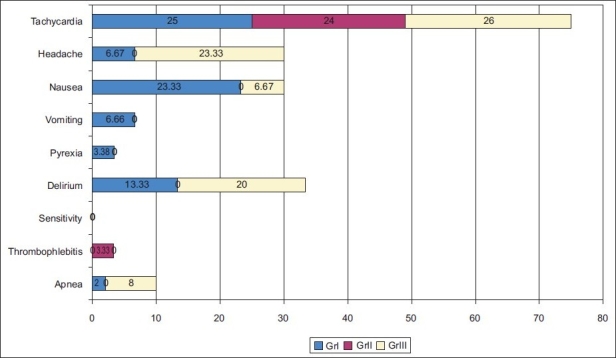
Incidence of side effects and complication after anaesthesia

Incidence of headache, nausea, vomiting, pyrexia and delirium were maximum with Group I and minimum with Group II, but incidence of pain on injection and thrombophlebitis were highest in Group II [Figure 3].

## DISCUSSION

ECT is one of the most widely recognised, accepted and most-effective treatment modality for various psychiatric illnesses. With the use of succinylcholine in 1951 by Wanderdel modified ECT came into existence. It reduces incidence of physical and psychological trauma.[3]

In the present study, the dose used for induction was calculated according to the body weight which was adequate to reach the induction criteria i.e. loss of eyelid reflex and could not interfere with the ECT induced seizure.

In the present study, induction was rapid with propofol as compared to thiopentone and midazolam, which was statistically significant (*P* < 0.05). Midazolam group had significant longer induction time than thiopentone and propofol (*P* < 0.001) which correlates well with the studies of various authors.[6,7]

Induction was smooth with propofol in comparison to thiopentone and midazolam. In the present study, high incidence of gag reflex coughing, tearing, movement of limbs was observed in thiopentone and midazolam groups compared to propofol. This finding was well correlated with observation of previous studies.[6–8]

Mean seizure duration was significantly shorter with midazolam followed by propofol and thiopentone group. Though significant shortening of seizure duration was observed with propofol which was above 25 seconds, it does not affect modified ECT efficacy or therapeutic outcome. Duration of seizure activity lasting for >25 sec in single session or a maximum of 210 sec of cumulative duration leads to good therapeutic outcome.[9] Elevation of seizure threshold after Propofol administration may explain the lower duration of seizure.

Increase in heart rate, SBP and DBP after ECT was observed in all the three groups but it was statistically highly significant in thiopentone group. Propofol and midazolam blunt the sympathetic response, so there was less increase in HR, SBP and DBP. The significant rise in HR after ECT with thiopentone as compared to midazolam and propofol was also noted by Boey WK *et al*,[5] Arya A *et al*[6] and Singhal SK *et al*.[8] In this study propofol seems superior to thiopentone in attenuating the physiological response to ECT with minimal haemodynamic changes. Arrhythmias occurred only in 6.67% patients in thiopentone group, which were transient and resolved spontaneously.

There was increase in serum potassium level in all the three groups after ECT which was not statistically significant. This may be due to synchronous contraction of all the voluntary muscles of body which are rich in potassium secondary to succinylcholine induced fasciculations which pump out potassium into general circulation causing a greater increase in plasma potassium concentration. Bali IM[10] and Agarwal R[11] also found increase in serum potassium after modified ECT.

A significant difference in recovery time was observed among the groups. Propofol group had significantly earliest and smooth recovery (*P* < 0.05), followed by thiopentone and midazolam with respect to time for the ability to obey verbal command, ability to sit unaided. The mean time taken to meet discharge criteria was also significantly (*P* < 0.05) high in midazolam group (22.25 min) and least in propofol group (11.59 min) similar to other studies. An early recovery helps in early discharge to home.[5,6,8]

The incidence of nausea and vomiting was almost nil in propofol due to antiemetic property as compared to 23.33% in thiopentone and 6.67% in midazolam. 20% patients complained of pain on injection and 3.33% patients had thrombophlebitis with propofol compared 0% with thiopentone and midazolam. That can be easily attenuated by using 1% lignocaine or by using large veins for injection.[6–8]

## CONCLUSION

To conclude propofol in the dosage of 2 mg/kg body weight intravenously can be safely used for modified ECT in ASA Grade I and II patients. Fast, smooth induction, early smooth recovery, better haemodynamics, antiemetic property, uncompromised therapeutic outcome without much rise in serum potassium makes propofol as an agent of choice for day care procedure.
